# Diagnostic Pitfalls and Management of Transphyseal Fractures of the Distal Humerus: A Retrospective Review of 25 Cases

**DOI:** 10.3390/children13030352

**Published:** 2026-02-28

**Authors:** Li Zhang, Yang Yuan, Haoqi Cai, Yufeng Wang, Yuchan Li, Haiqing Cai, Zhigang Wang, Mingyuan Miao

**Affiliations:** 1 Department of Pediatric Orthopedics, Shanghai Children’s Medical Center, Shanghai Jiao Tong University School of Medicine, Shanghai 200127, China; zhangli@scmc.com.cn (L.Z.);; 2 Department of Anesthesiology, Shanghai Children’s Medical Center, Shanghai Jiao Tong University School of Medicine, Shanghai 200127, China

**Keywords:** transphyseal fractures of the distal humerus, pediatric elbow fracture, physeal separation, diagnostic errors, cubitus varus

## Abstract

**Background/Objectives**: Transphyseal fracture of the distal humerus (TFDH) is a rare but clinically important pediatric elbow injury that predominantly affects children under 3 years of age. Due to the radiolucent nature of the cartilaginous distal humeral epiphysis in this age group, TFDH is often misdiagnosed as elbow dislocation, supracondylar fracture, or lateral/medial condyle fracture. Time pressures, limited pediatric musculoskeletal expertise, and incomplete clinical histories in emergency settings further compound this diagnostic challenge. Despite the importance of early and accurate diagnosis to prevent complications such as cubitus varus, systematic studies on diagnostic pitfalls and strategies for improving recognition remain scarce. We therefore aim to characterize misclassification patterns, standardize radiographic cues, and evaluate management outcomes. **Methods**: We conducted a single-center retrospective review of 25 pediatric patients with TFDH who were misdiagnosed at initial presentation between 2012 and 2022. Clinical records, radiographic features, treatment modalities, and complications were analyzed over a minimum follow-up period of 24 months. **Results**: All 25 cases were initially misdiagnosed. The most common misdiagnoses were supracondylar and lateral condyle fractures (each 6/25, 24%), followed by elbow dislocation (4/25, 16%). Misclassification was primarily attributed to failure to assess global forearm–humerus alignment and misinterpretation of the radiocapitellar line. All patients underwent emergency management, with 18/25 (72%) receiving closed reduction and percutaneous K-wire fixation, and 7/25 (28%) undergoing closed reduction and cast immobilization. Cubitus varus developed in 5/25 (20%) overall and was more frequent after closed reduction with cast immobilization (3/7, 43%) than after K-wire fixation (2/18, 11%). Overall, 92% achieved excellent functional outcomes according to the Mayo Elbow Performance Index (MEPI). The implementation of a targeted curriculum improved diagnostic accuracy among trainees from 70% to 100%. **Conclusions**: TFDH poses substantial cognitive and radiographic diagnostic challenges. A structured radiographic assessment, early senior review, and targeted education may improve recognition and outcomes. These findings offer actionable insights to enhance diagnostic accuracy and optimize care for this vulnerable patient population.

## 1. Introduction

Transphyseal fracture of the distal humerus (TFDH) is a rare pediatric elbow injury that predominantly affects infants and toddlers under 3 years of age [1]. Reported proportions vary by series; in neonates, the condition is exceedingly uncommon and occurs less than 0.00001% [2,3]. While specific misdiagnosis rates for TFDH are not uniformly reported across large cohort studies, the available literature consistently highlights it as an “often-missed” fracture. One recent retrospective study reported approximately 50% misdiagnosis rates of TFDH in the emergency department [4]. Such high error rates can delay definitive treatment and subsequent development of malunion, potentially leading to malunion, osteonecrosis, growth disturbance, progressive angular deformity (classically cubitus varus), and eventually increased need for surgical corrections. By contrast, other common pediatric elbow injuries, such as supracondylar and radial head fractures, generally demonstrate greater remodeling potential and are more likely to achieve favorable functional outcomes [5].

In very young children, the distal humeral epiphysis is largely cartilaginous and radiolucent, which obscures direct fracture lines on plain radiographs and facilitates misclassification as elbow dislocation, supracondylar fracture, lateral/medial condyle fractures, or it can be missed completely [6]. This diagnostic challenge is magnified in emergency settings, where time pressure, limited pediatric musculoskeletal expertise, and reliance on incomplete caregiver histories may amplify cognitive and perceptual biases—particularly among junior clinicians. Furthermore, young children often present with nonspecific symptoms and limited cooperation, while unwitnessed injuries or abuse-related mechanisms further cloud clinical interpretation. These subtleties also underline common diagnostic confounders, including lateral or medial condyle fractures with an overlooked physeal component, supracondylar fractures of the distal humeral metaphysis, and Monteggia lesions with true radiocapitellar disruption. Additionally, non-traumatic mimics such as septic arthritis, nursemaid’s elbow, and neonatal brachial plexus palsy may present with pseudoparalysis or limited use, further broadening the differential diagnosis in early childhood. Additionally, non-accidental injury (NAI)—including birth-related trauma and child abuse—should also be considered in suspicious cases [7]. Given the rarity of TFDH and its prevalence in very young children, NAI—including child abuse—must be a key consideration in the differential diagnosis. A thorough evaluation of the injury mechanism and clinical history is critical, and any suspicion of NAI requires the immediate involvement of a child protection team to ensure the safety and well-being of the child.

Management strategies for TFDH remain variable, spanning conservative immobilization to surgical fixation. While early childhood affords substantial remodeling potential, nonoperative treatment carries a meaningful risk of residual deformity [8]. Ultrasound- or arthrography-guided closed reduction with internal fixation has yielded satisfactory radiographic and functional outcomes, albeit with complications such as pin migration and cubitus varus [9]. In practice, closed reduction and percutaneous pinning (CRPP) and open reduction with internal fixation (ORIF) [10] have been used for TFDH. Extrapolating from broader pediatric upper-limb fracture literature, supplemental pinning reduces redisplacement and better maintains alignment at the expense of pin-related complications [11]. Yet TFDH-specific data remain scarce. To address these gaps, we conducted a single-center, two-year retrospective review of 25 TFDH cases initially misidentified in emergency departments. We examined clinical records, radiographs, intraoperative arthrography, and classified injuries using the modified DeLee classification. We analyzed mechanisms of injury, initial diagnostic labels, and radiographic features to delineate cognitive and imaging pitfalls, and we assessed operative strategies and outcomes during follow-up. Our aim is to provide a concise, evidence-informed framework to improve diagnostic accuracy, expedite definitive care, and enhance patient safety in this vulnerable population.

## 2. Materials and Methods

This was a single-center retrospective review conducted at the Department of Pediatric Orthopedics, Shanghai Children’s Medical Center. The study period spanned January 2012 to December 2022. This study was approved by the Institutional Review Board of Shanghai Children’s Medical Center affiliated with Shanghai Jiao Tong University (SCMCIRB-K2024118-1). Written informed consent was obtained from the parent or legal guardian of each participant. All data were de-identified before analysis, and the study adhered to the principles of the Declaration of Helsinki.

### 2.1. Patient Selection

Inclusion criteria were: (1) definitive diagnosis of TFDH confirmed by senior radiographic review and/or intraoperative arthrography; (2) availability of complete clinical and radiographic data; (3) documentation of an initial alternative diagnosis (e.g., supracondylar fracture, lateral/medial condyle fracture, elbow dislocation) at the first encounter; (4) minimum clinical follow-up of 24 months, as progressive worsening of varus alignment beyond 24 months is exceedingly rare.

Exclusion criteria: (1) incomplete clinical or imaging records; (2) pathological fractures; (3) concomitant ipsilateral long-bone injuries that precluded reliable assessment of elbow alignment; (4) patients referred from other hospitals with a prior diagnosis of TFDH.

### 2.2. Data Collection

Demographic and clinical variables included age, sex, side, mechanism of injury (ground-level fall, fall from height, suspected non-accidental injury), time from injury to presentation, and referring facility. We recorded initial ED diagnostic labels and the clinician’s level of training (years of independent practice). Misclassification at initial presentation was attributed when the first documented diagnosis by the ER orthopedic physician differed from the final TFDH diagnosis.

### 2.3. Imaging Acquisition and Assessment

Anteroposterior (AP) and lateral elbow radiographs were obtained at presentation. Two pediatric orthopedic surgeons (each with ≥10 years of experience) independently reviewed all imaging to record alignment parameters (radiocapitellar alignment, ulnohumeral congruity, en bloc epiphysis–forearm translation/rotation, directionality). Fractures were classified using a modified DeLee classification (types A–C) [12]. Disagreements were resolved by consensus. Associated injuries recorded included medial or lateral condyle components suggestive of a physeal fragment (e.g., a Thurston–Holland fragment), ulnar fractures (to evaluate for Monteggia patterns), and the presence of anterior or posterior fat pad signs. Cubitus varus was defined as a visible loss of the carrying angle, characterized by a humeroulnar angle measuring less than 0 degrees. This deformity results in the forearm deviating medially when the arm is fully extended, often referred to as a “gunstock deformity”.

### 2.4. Treatment and Follow-Up

All patients received emergency management on the same day or the day following their presentation to the emergency department. Treatment modalities included closed reduction with percutaneous K-wire fixation and closed reduction with cast immobilization. Post-reduction alignment was verified radiographically. Pin care and cast management followed institutional protocols. Patients were followed for a minimum of 24 months, with scheduled assessments of alignment, range of motion, complications (including cubitus varus), and functional outcomes using the Mayo Elbow Performance Index (MEPI). Adverse events, reinterventions, and any neurovascular complications were recorded.

### 2.5. Targeted Curriculum Detail

A curriculum was designed to address common diagnostic challenges encountered by trainees in the management of pediatric elbow fractures. It consisted of structured educational sessions, including lectures, case-based discussions, and hands-on workshops. The lectures covered key topics such as the anatomy of the pediatric elbow, common fracture patterns, diagnostic imaging interpretation, and clinical decision-making algorithms. Case-based discussions focused on real-world scenarios, enabling trainees to apply theoretical knowledge to practical situations. The hands-on workshops included supervised practice in interpreting radiographs and performing diagnostic assessments. Senior orthopedic surgeons facilitated the sessions, ensuring high-quality instruction and guidance.

### 2.6. Outcomes and Statistical Analysis

Primary outcomes were patterns of initial misclassification and radiographic contributors to misdiagnosis. Secondary outcomes included treatment distribution, complication rates (with a focus on cubitus varus), and functional results (MEPI), which use a 100-point scale and classify scores as excellent (≥90), good (75–89), fair (60–74), or poor (<60). Descriptive statistics are reported as absolute numbers and percentages. Analyses were performed using IBM SPSS Statistics, Version 26.0 (IBM Corp., Armonk, NY, USA).

## 3. Results

### 3.1. Cohort Characteristics

A total of 63 patients were diagnosed with transphyseal fractures of the distal humerus (TFDH) during the study period. Among these, 25 cases (40.3%) were initially misdiagnosed and thus were included. The mean age at the time of injury was 27 ± 10.8 months (range: 11–56 months). The most common injury mechanism was ground-level falls (16/25, 64%), followed by falls from height (7/25, 28%), being fallen-on (1/25, 4%), and one case of suspected non-accidental injury (1/25, 4%).

### 3.2. Radiographic Classification and Displacement

Based on the modified DeLee classification, injuries were categorized as type A in 2 cases (8%), type B in 16 cases (64%), and type C in 7 cases (28%). The initial displacement direction was ulnar in 15 cases (60.0%), radial in 3 cases (12.0%), and indeterminate in 7 cases (28.0%).

### 3.3. Initial Diagnosis and Misclassification

**Figure 1:**
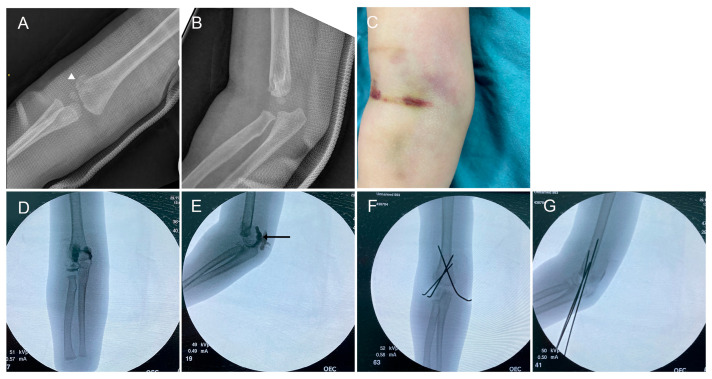
Transphyseal distal humerus fracture (TFDH) misdiagnosed as a lateral condyle fracture. A 17-month-old girl (Case 17) was initially misdiagnosed with a lateral condyle fracture. (**A**,**B**): Initial anteroposterior and lateral radiographs. White arrowhead denotes the metaphyseal (Thurston–Holland) fragment. (**C**): Soft tissue swelling of both the medial and lateral aspects of the elbow is present. (**D**,**E**): Arthrograms show posterior displacement of the distal humeral epiphysis with intact articular cartilage (black arrows). (**F**,**G**): Closed reduction and percutaneous pin fixation performed under arthrography guidance.

**Figure 2:**
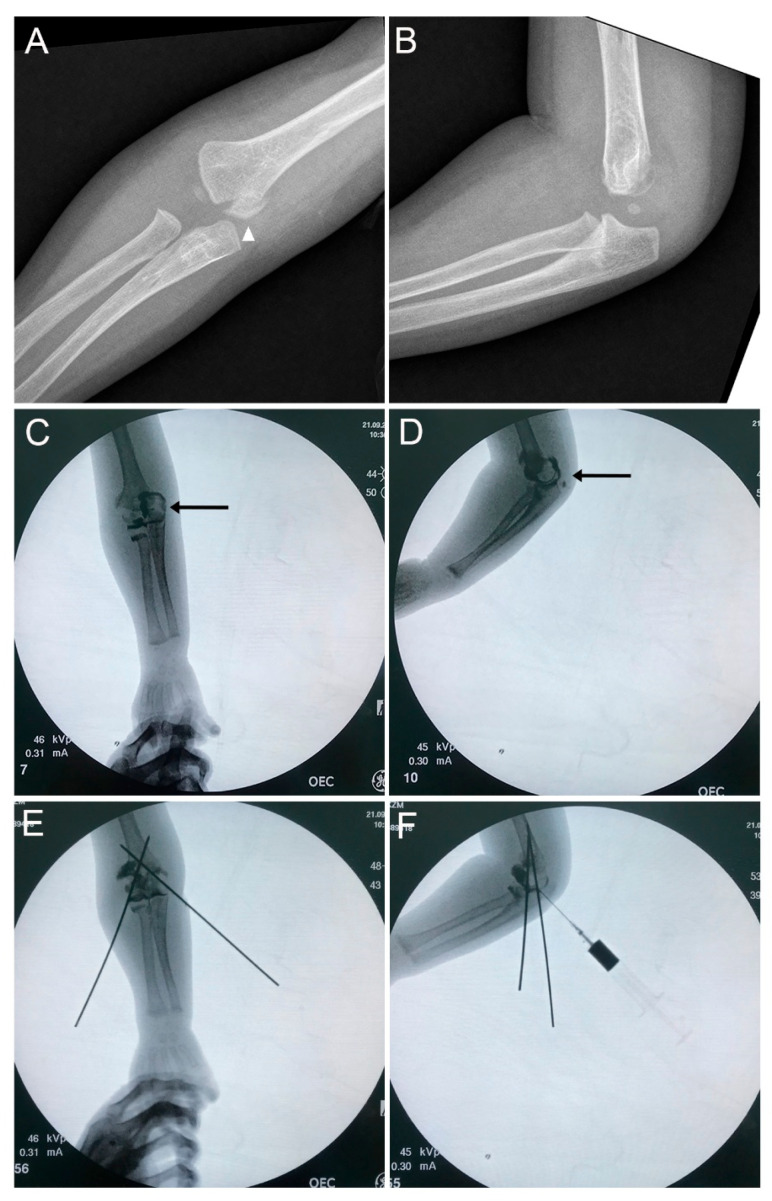
Transphyseal distal humerus fracture (TFDH) misdiagnosed as a medial condyle fracture. A 12-month-old boy (Case 12) was initially misdiagnosed with a medial condyle fracture. Soft-tissue swelling of the both medial and lateral elbow is present. (**A**,**B**): Initial anteroposterior and lateral radiographs. White arrowhead denotes the metaphyseal fragment (Thurston–Holland sign). (**C**,**D**): Arthrograms show posteromedial displacement of the distal humeral epiphysis with intact articular cartilage (black arrows). (**E**,**F**): Closed reduction and percutaneous pin fixation performed under arthrography guidance.

**Figure 3:**
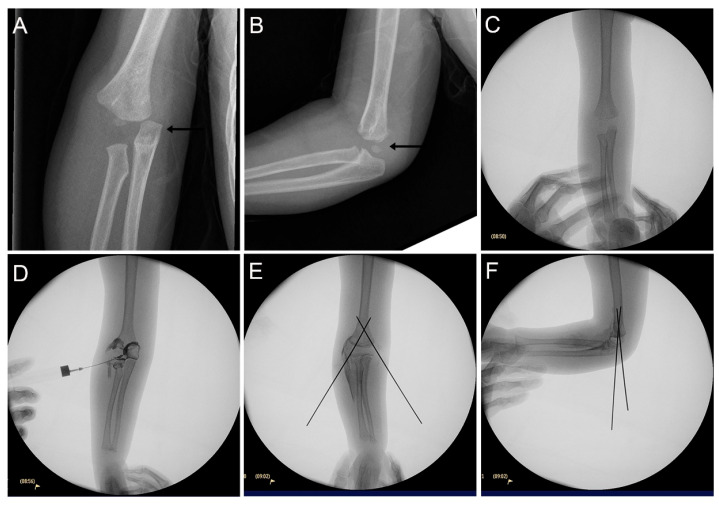
Transphyseal distal humerus fracture (TFDH) misdiagnosed as elbow dislocation. A 29-month-old girl (Case 18) was initially misdiagnosed with an elbow dislocation. (**A**,**B**): Initial anteroposterior and lateral radiographs show posteromedial displacement of the forearm relative to the distal humerus (black arrows); Note the capitellum is not yet ossified. (**C**–**F**): Closed reduction and percutaneous pin fixation performed under arthrography guidance.

None of the cases were correctly identified as TFDH at initial presentation. Preliminary diagnoses varied widely, including lateral condyle fracture (LCF) in 5 cases (20%) (Figure 1), medial condyle fracture (MCF) in 3 cases (12%) (Figure 2), MCF with elbow dislocation in 3 cases (12%), LCF with elbow dislocation in 1 case (4%), supracondylar fracture (SCF) in 6 cases (24%), and isolated elbow dislocation in 5 cases (20%) (Figure 3). Two patients were diagnosed as “normal elbow” and “nursemaid’s elbow”, respectively.

Of the 25 cases analyzed, the leading sources of misclassification were overlooking global forearm–humerus alignment (36%), misinterpretation of the radiocapitellar line (32%), misreading metaphyseal fragments as evidence of SCF (24%), overreliance on a visible fracture line as a “must-have” (4%), and misleading information given by caregiver (4%). Misclassification clustered by initial labels: cases initially labeled as “elbow dislocation” or “MCF/LCF + dislocation” predominantly stemmed from radiocapitellar line errors; those labeled “MCF/LCF” were mainly driven by neglect of global alignment; and those labeled “SCF” concentrated in the SCF misread category. Recorded displacement directions frequently included ulnar/radial components consistent with the characteristic posteromedial translation pattern of TFDH, yet these cues were missed when views were suboptimal or systematic alignment checks were not performed.

### 3.4. Targeted Curriculum for Diagnostic Accuracy

The implementation of the targeted curriculum resulted in a significant improvement in diagnostic accuracy among trainees. Prior to the curriculum, diagnostic accuracy was measured at 70%. Following the completion of the curriculum, diagnostic accuracy increased to 100%.

### 3.5. Treatment

All patients underwent urgent operative care; no open reduction and internal fixation was required. CRPP was performed in 18/25 (72%), and closed reduction with casting was performed in 7/25 (28%). Casting was used more frequently in type B injuries, whereas type C patterns were more commonly treated with CRPP.

### 3.6. Outcomes and Complications

Two patients experienced delayed wound healing with superficial infection, both successfully managed nonoperatively. No cases of neurovascular injury or deep infection were reported. Over a minimum follow-up period of 24 months, cubitus varus developed in 5 patients (20.0%), occurring in 3 of 7 closed reduction with casting cases (42.9%) and 2 of 18 CRPP-treated cases (11.1%). Functional outcomes by the MEPI were predominantly excellent, with 23/25 (92%) graded as excellent. All patients achieved a sufficient range of motion to fully participate in age-appropriate daily and sports activities without discomfort. A comprehensive summary of demographic, radiographic, and clinical data is presented in Table 1.

## 4. Discussion

Accurate diagnosis of TFDH in the ED remains a significant challenge. In our study, the initial misdiagnosis rate is 40.3%, similar to the previously reported rate [4]. These findings highlight the significant prevalence of initial diagnostic errors in clinical practice. This high misdiagnosis rate in our study not only reinforces the notion that TFDH is frequently overlooked during early clinical encounters, but also reflects the significant challenges faced by young clinicians, particularly in emergency settings where time pressures and pediatric expertise are limited. Among the 26 initially misdiagnosed cases, only one was diagnosed as normal elbow, and the other 25 cases were misclassified as supracondylar fractures, elbow dislocations, or condylar fractures. These results strongly indicate that TFDH is not simply a rare pediatric elbow injury but a diagnostically dangerous pathology that often masquerades as a more common elbow injury. Notably, the majority of diagnostic errors occurred among junior clinicians within their first two years of practice. In contrast, cases initially evaluated by senior staff in our series were diagnosed with 100% accuracy. Among junior clinicians, overconfidence combined with limited familiarity with the radiographic appearance likely contributed to misinterpretation of equivocal radiographs and misclassification as more familiar entities (e.g., supracondylar or lateral condyle fractures, elbow dislocation, or “no fracture”). Importantly, several senior surgeons in our institution acknowledged having misdiagnosed TFDH during their own early ED experience, highlighting that clinical expertise and pattern recognition are critical determinants of diagnostic accuracy. Our findings align with prior literature indicating that diagnostic performance in pediatric outpatient acute care differs by practice level, and senior specialists make significantly fewer diagnostic errors than trainees [13,14]. However, these observations also suggest that experience alone may be insufficient to mitigate diagnostic errors in acute pediatric settings and underscore the need for targeted system-level interventions (targeted education, mentorship, and heightened awareness of TFDH).

Communication and contextual barriers play a critical role in the diagnostic challenges of TFDH. Due to pain and fear, young patients often struggle to localize pain, articulate injury mechanisms, or cooperate during physical examination. Furthermore, when injuries are unwitnessed, caregivers may provide incomplete, vague, or misleading histories, further complicating clinical assessment. In our series, one caregiver described the injury mechanism as a “twist” rather than a fall, leading to an initial diagnosis of soft-tissue injury. In another case, the caregiver reported only a “minor pull” to the elbow, offered an imprecise timeline, and initially refused radiographic evaluation. The child was provisionally diagnosed with nursemaid’s elbow. However, upon re-examination by senior clinicians, focal swelling and tenderness around the elbow were noted, along with fingertip-pattern ecchymoses on the child’s back and retroauricular regions—features discordant with the stated mechanism. Subsequent imaging confirmed TFDH, and the case was ultimately classified as non-accidental injury, triggering child protection involvement and definitive surgical intervention. These cases highlight the diagnostic pitfalls inherent in pediatric emergencies, where communication barriers and caregiver-related factors may bias clinical reasoning toward more familiar or benign diagnoses. Maintaining a high index of suspicion when physical findings and caregiver accounts diverge is essential. A meticulous physical examination, recognition of subtle injury patterns, and critical appraisal of history are vital to avoid misdiagnosis and ensure timely, appropriate intervention.

The observed misclassification patterns reveal a convergence of cognitive biases and procedural gaps in pediatric elbow fracture diagnosis. The most frequent error—neglecting global forearm–humerus alignment—suggests that clinicians may overly focus on localized fracture features, overlooking broader joint translation cues. Fragment-centric bias draws attention to medial or lateral condyle fragments at the expense of assessing overall alignment; misapplication of the radiocapitellar line leads to erroneous diagnoses of elbow dislocation—particularly problematic in toddlers, where true dislocation is exceedingly rare; and visibility bias promotes the absence of a clear cortical break as a false negative for TFDH.

These data underscore a critical need for systematic assessment protocols in pediatric elbow trauma. In response, we developed and implemented a focused, competency-based curriculum emphasizing age-specific diagnostic triggers, radiographic alignment analysis, and clinical context. By translating key diagnostic differentiators into actionable workflows and embedding an age-based trigger (<3 years) alongside a mandatory two-step alignment check (global forearm–humerus translation and radiocapitellar alignment), the program elevated diagnostic accuracy from 70% to 100% among trainees. This underscores the value of targeted, competency-based education for early-career physicians [15,16,17,18]. The following numbered framework codifies these steps.

Age-Based Diagnostic Trigger: TFDH should be considered a mandatory differential diagnosis in all children under 3 years of age presenting with elbow swelling and focal distal humeral tenderness, particularly when the mechanism of injury or clinical findings suggest potential physeal involvement. Given the rarity of true elbow dislocation in this age group, any diagnosis of elbow dislocation warrants careful reassessment and a high index of suspicion for TFDH.

Signature Radiographic pattern of TFDH: TFDH involves a shear through the distal humeral physis, often radiographically occult. Diagnosis hinges on indirect signs: en bloc posteromedial (occasionally posterior or posterolaterally) translation of the radius and ulna relative to the metaphysis, with preserved proximal radioulnar alignment. Ulnohumeral congruity is disrupted, while the radiocapitellar line remains intact if the capitellum is ossified. Ultrasound is the indispensable point-of-care imaging tool for suspected TFDH. In TFDH, ultrasound can directly visualize the disruption of the physis, displacement of the unossified epiphyseal fragment relative to the metaphysis, and periosteal sleeve integrity. A joint effusion or hemarthrosis is a key finding, indicating intra-articular breach. MRI is the gold standard imaging modality for TFDH patients, providing unparalleled visualization of the physeal disruption, unossified epiphyseal fragment displacement, bone marrow edema, and associated soft tissue findings.

Elbow dislocation (true vs. TFDH): Both TFDH and true elbow dislocation may show ulnohumeral incongruity. The key discriminator is the radiocapitellar relationship. In TFDH, the radial head displaces together with the humeral epiphysis, preserving radiocapitellar alignment; true dislocation disrupts it due to ligamentous failure without epiphyseal separation. Because the physis is weaker than ligaments in infants/toddlers, true dislocation is exceedingly rare under 3 years of age [7]. Any diagnosis of elbow dislocation in this age group warrants careful reassessment and a high index of suspicion for TFDH (Figure 1). Ultrasound excels at differentiating TFDH from true posterior elbow dislocation, which disrupts ulnohumeral congruity but preserves the physis.

Condyle fractures with “elbow dislocation”: In addition to four cases recorded as isolated elbow dislocation, our data highlights three cases documented as “MCF with elbow dislocation” and one “LCF with elbow dislocation” with the physeal injury overlooked (Figure 2 and Figure 3). This pattern reflects a composite appearance in toddlers: global distal fragment translation/rotation that mimics dislocation coexisting with medial/lateral condyle fragment (Thurston–Holland sign), which masks the transphyseal nature of the injury. In isolated condyle fractures, global forearm-to-humerus alignment is preserved; in TFDH it is not. Practically, any initial label of “MCF + elbow dislocation” or “elbow dislocation” should trigger a TFDH checklist: verify ulnohumeral congruence relative to the metaphysis; obtain contralateral comparison views; use point-of-care ultrasound to demonstrate physeal discontinuity; reserve MRI for equivocal or late presentations. Then, use point-of-care ultrasound to demonstrate physeal discontinuity, and reserve MRI for equivocal cases.

SCF vs. TFDH: Both may demonstrate posterior displacement of the distal fragment and preservation of the radiocapitellar line. SCF traverses the distal humeral metaphysis and typically shows a visible cortical break; fat pad signs (anterior/posterior) increase confidence when the break is subtle. TFDH is an epiphysis that separates from the metaphysis through the growth plate and may lack a discernible fracture line unless accompanied by medial or lateral condyle involvement.

Monteggia fracture: Monteggia lesions, more common in children aged 4–10, involve ulnar fracture with radial head dislocation and disrupted radiocapitellar alignment, with loss of proximal radioulnar congruity. In contrast, TFDH presents with intact ulna and preserved radiocapitellar congruity due to en bloc displacement, while proximal radioulnar alignment remains intact.

Septic arthritis: Both conditions may present with pseudoparalysis in infants. Septic arthritis is typically preceded by systemic symptoms and elevated inflammatory markers, with ultrasound revealing joint effusion. TFDH lacks systemic signs and presents with focal tenderness. MRI can be applied to distinguish marrow edema and synovial enhancement (infection) from physeal disruption (TFDH).

Nursemaid’s elbow: Common in toddlers after axial traction, nursemaid’s elbow presents with refusal of supination and minimal swelling or tenderness. TFDH, in contrast, involves trauma history, elbow swelling, and focal tenderness. Imaging is warranted when history or exams are atypical.

**Figure 4:**
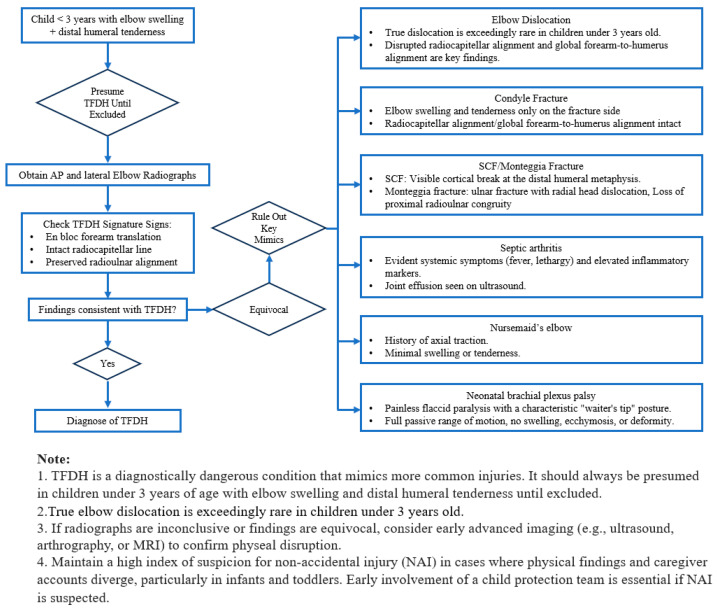
Diagnostic Flowchart for Transphyseal Fractures of the Distal Humerus (TFDH) in Children Under 3 Years of Age.

Neonatal brachial plexus palsy (NBPP): NBPP and TFDH may both follow difficult deliveries. NBPP presents with painless, flaccid paralysis, full passive range of motion, and a normal limb contour, while TFDH involves pain on manipulation, focal swelling or ecchymosis and restricted passive range of motion. NBPP typically affects the upper trunk (C5-C6), leading to Erb’s palsy, which presents with the classic “waiter’s tip” posture: the arm is adducted and internally rotated at the shoulder, the elbow is extended, the forearm is pronated, and the wrist is flexed. Plain radiographs become essential when the clinical picture is ambiguous. Radiographs may be inconclusive in both cases. Ultrasound and arthrography may be required. In such cases, point-of-care ultrasound has emerged as a valuable tool to detect occult physeal separation that is not apparent on X-ray [19]. MRI is used primarily to assess the brachial plexus itself but not recommended for initial differentiation from fracture [20]. A diagnostic flowchart in children under 3 years of age is presented (Figure 4).

Early and accurate diagnosis of TFDH is unequivocally critical for ensuring timely management and preventing a spectrum of serious complications [4]. However, the complexity of the ER environment and the cartilaginous nature of the distal humeral epiphysis often lead to misinterpretation and insufficient evidence for diagnosing TFDH. This highlights the critical need for early application of additional imaging modalities, if available, to prevent delays in diagnosis and treatment, which can otherwise lead to complications such as deformities and growth disturbances. Ultrasound serves as a rapid, first-line imaging modality, offering real-time visualization of physeal disruption, epiphyseal displacement, and periosteal sleeve integrity while differentiating TFDH from elbow dislocations. Arthrography, though used less frequently, can confirm intra-articular fractures and delineate cartilaginous fragments with the aid of contrast. MRI, as the gold standard, provides comprehensive visualization of the unossified cartilaginous epiphysis, physeal plate, joint capsule, ligaments, and surrounding structures, enabling precise characterization of the injury and differentiation from other pediatric elbow pathologies such as supracondylar fractures, condylar fractures, and septic arthritis. The early and appropriate use of these imaging modalities, particularly when radiographs are inconclusive, is critical to ensure accurate diagnosis, timely management, and prevention of long-term complications.

System-level supports, in addition to targeted education, played a pivotal role in enhancing diagnostic reliability in acute pediatric settings. Real-time and follow-up consultations with senior staff provided immediate corrective feedback, while structured next-day handover reviews of flagged or uncertain elbow injuries acted as a crucial safety net. In our cohort, misdiagnoses were identified and corrected by senior doctors either on the same day of presentation or the following day after the patient was handed over. A notable example is Patient 9, who presented at 2 a.m. and was initially assessed as having a “normal elbow,” highlighting the subtlety of radiographic findings and the potential for complete diagnostic oversight. However, during the morning meeting, a senior doctor identified the error and promptly corrected the diagnosis. These collaborative discussions among medical teams not only improved diagnostic precision but also fostered shared learning about the complexities of TFDH. Such system-level measures are essential to reduce misdiagnoses in this vulnerable population and ensure optimal patient outcomes.

TFDH is strongly associated with non-accidental injury. Previous clinical evidence demonstrates that TFDH is commonly linked to abusive trauma in this vulnerable population [21]. Given the high stakes involved—both for the immediate safety of the child and the potential legal and social ramifications for the family—a systematic, multidisciplinary approach to diagnosis and management is not merely advisable but mandatory. A thorough history should include detailed accounts of the injury mechanism, timing, and any inconsistencies in caregiver reports. Injuries resulting from unwitnessed incidents or mechanisms inconsistent with the child’s developmental stage (e.g., a fall from a height in a non-ambulatory infant) should raise suspicion [22]. Second, a complete head-to-toe examination should be conducted to assess for other signs of trauma, such as bruising, burns, or fractures at different stages of healing. Special attention should be given to the presence of swelling, tenderness, or deformity in untypical areas of the body. Third, a skeletal survey should be performed in all suspected cases of NAI to identify other fractures or injuries that may not be clinically apparent. Additional imaging modalities such as MRI or CT may be considered to evaluate intracranial or visceral injuries. Fourth, in cases of suspected NAI, prompt referral to a multidisciplinary child protection team is essential. This team typically includes pediatricians, social workers, and law enforcement professionals who can conduct a thorough investigation and ensure the safety of the child [23]. Furthermore, all findings, including clinical observations, imaging results, and caregiver accounts, should be meticulously documented to support any necessary legal proceedings. We recommend that all children presenting with TFDH undergo an initial evaluation by a senior pediatric orthopedic surgeon, with mandatory notification of the child protection team in cases of suspected NAI. There is currently no universally accepted treatment protocol for TFDH. Management options in young children include closed reduction with immobilization, CRPP, and ORIF. In neonates, due to substantial remodeling capacity and rapid healing, many authors advocate initial conservative treatment—typically gentle closed reduction followed by cast—when post-reduction alignment is satisfactory and stability is maintained [7,24,25]. However, inadequate reduction or instability may predispose to secondary displacement and subsequent cubitus varus [24].

The choice of treatment (Casting versus CRPP) was based on a synthesis of radiographic, clinical, and biological factors, as assessed by the attending orthopedic surgeon. In our cohort, CRPP was typically performed for displaced fractures, while casting was used for minimally displaced or stable fractures where a satisfactory alignment can be maintained [1]. The overall incidence of cubitus varus was 16%, occurring significantly more frequently in the casting group (2/7, 28.6%) than in the CRPP group (2/16, 12.5%). The primary cause of cubitus varus in conservatively treated children was loss of reduction. Plaster immobilization alone failed to provide sufficient stability, and serial radiograph review confirmed that displacement occurred during the immobilization period. In contrast, CRPP demonstrated a lower rate of cubitus varus, though two cases of pin tract infection were noted. External comparative studies report lower residual deformity rates and superior functional outcomes following ORIF [10] compared to conservative care. This trend is echoed in literature on other upper limb fractures, where additional pinning is associated with reduced re-displacement and functional limitation, albeit with increased risks of complications such as pin tract infection and neurovascular injury [26,27]. Despite these differences, our data indicated that the mean range of motion appeared comparable between the casting and CRPP groups. These findings suggest that while functional outcomes may appear comparable in the short term, nonoperative management may yield suboptimal results due to instability and loss of reduction, even in children with theoretical remodeling potential.

The incidence of cubitus varus was higher in the Casting group (43%) compared to the CRPP group (11%), but given the small sample sizes in these subgroups, this difference may be due to chance and should be interpreted with caution. Among the CRPP group, two children (patients 20 and 23) developed cubitus varus. For patient 23, a treatment delay of approximately four days between the injury and presentation may have contributed to deformity and compromised function, consistent with prior reports linking delayed intervention to poorer outcomes [28,29]. Another plausible factor is suboptimal coronal reduction and fixation: In very young children, the distal humeral epiphysis remains largely cartilaginous and unossified, making plain radiographs unreliable for assessing alignment. In patient 20, surgery was performed without intraoperative arthrography or ultrasonography, limiting the ability to achieve precise reduction and stable fixation [9,30], thereby increasing the risk of malunion and varus deformity. Additional contributors may include physeal injury inherent to TFDH (Salter–Harris type I or II), which can lead to growth disturbance or medial condyle bony bar formation [6]. Iatrogenic factors—such as repeated forceful reduction attempts, excessive traction, or pin-related compression—may further compromise the tenuous blood supply, elevating the risk of AVN and subsequent cubitus varus [31].

To our knowledge, this is the first study to systematically examine the diagnostic pitfalls and initial evaluation of TFDH in a pediatric emergency setting. By analyzing misclassification patterns, radiographic features, and clinical context across a sizable cohort, we provide novel insights into the subtle and frequently overlooked presentation of TFDH in young children. These findings fill a critical gap in the pediatric orthopedic literature and offer a practical framework for improving early diagnosis and management of this rare but impactful injury, ultimately enhancing patient safety and long-term recovery.

This study has several limitations. First, its retrospective, single-center design introduces inherent selection, information, and documentation biases. Although 25 cases represent a relatively large cohort for such a rare injury, the sample size remains modest. As a result, statistical power for subgroup analyses—such as comparisons by modified DeLee classification, injury mechanism, or specific misdiagnosis categories—is limited, and the precision of complication rate estimates is reduced. Larger, multicenter studies are needed to validate these findings and improve external generalizability. Second, while physician-related diagnostic factors were examined, other potential contributors—such as clinician fatigue, sleep deprivation, high workload, and suboptimal radiographic quality—were not assessed. These factors may have influenced diagnostic performance and should be considered in future investigations. Finally, the role of adjunct imaging modalities such as ultrasound and MRI, which may enhance diagnostic certainty in equivocal cases, was not systematically evaluated in our cohort. Future studies should explore their diagnostic utility and integration into early assessment protocols for suspected TFDH.

## 5. Conclusions

TFDH poses substantial cognitive and radiographic diagnostic challenges and is therefore frequently misdiagnosed as more common elbow injuries. A structured radiographic assessment, early senior review, and targeted education may improve recognition and outcomes. We recommend that TFDH be considered a mandatory differential diagnosis in all children under 3 years of age presenting with elbow injuries in the ER. Furthermore, all children presenting with TFDH should undergo an initial evaluation by a senior pediatric orthopedic surgeon, with mandatory notification of the child protection team in cases of suspected NAI.

## Figures and Tables

**Table 1:** Summary of Diagnostic Radiograph Details of All Subjects.

Patient No.	Gender	Age (Months)	Injury Cause	Initial Diagnosis	Concomitant Injuries	Displacement Direction	Modified Delee’s Classification	Treatment	Time from Injury to Hospital Presentation (Hours)	Complications	MEP Score
1	Male	16	Falls on ground	LCF	/	Not obvious	B	CRPP	10	/	Excellent
2	Female	29	Fall from height	LCF	/	Not obvious	B	CRPP	7	Surgical site infection	Excellent
3	Male	56	Falls on ground	LCF with elbow dislocation	/	Not obvious	C	CRPP	3	/	Excellent
4	Male	37	Falls on ground	MCF with elbow dislocation	Ulnar nerve injury	Ulnar	C	CRPP	10	/	Excellent
5	Male	28	Fall from height	MCF with elbow dislocation	/	Ulnar	C	CRPP	8	/	Excellent
6	Male	24	Falls on ground	MCF with elbow dislocation	/	Ulnar	C	CRPP	9	/	Excellent
7	Male	39	Falls on ground	MCF	/	Not obvious	B	CRPP	2	Surgical site infection	Excellent
8	Female	35	Fall from height	SCF	/	Ulnar	C	CRPP	4	/	Excellent
9	Male	43	Falls on ground	Normal elbow	/	Ulnar	B	Cast	12	/	Excellent
10	Male	25	Fall from height	LCF	/	Radial	C	CRPP	6	/	Excellent
11	Female	11	Fall from height	MCF	Ulnar nerve injury	Ulnar	B	Cast	11	Cubitus varus	Good
12	Male	12	Others fallen on elbow	MCF	/	Ulnar	B	CRPP	5	/	Excellent
13	Female	45	Falls on ground	SCF	/	Not obvious	B	Cast	13	Cubitus varus	Excellent
14	Female	17	Falls on ground	SCF	/	Ulnar	B	Cast	14	/	Excellent
15	Male	23	Falls on ground	SCF	/	Not obvious	B	Cast	9	/	Excellent
16	Male	31	Fall from height	SCF	/	Ulnar	B	CRPP	7	/	Excellent
17	Female	17	Falls on ground	LCF	/	Radial	B	CRPP	10	/	Excellent
18	Female	29	Falls on ground	Elbow dislocation	/	Ulnar	B	CRPP	9	/	Excellent
19	Male	19	Falls on ground	SCF	/	Ulnar	B	CRPP	6	/	Excellent
20	Female	25	Fall from height	Elbow dislocation	/	Ulnar	B	CRPP	3	Cubitus varus	Good
21	Female	23	Suspected non-accidental trauma	Nursemaid’s elbow	Bruises in unusual locations, malnourishment	Not obvious	B	CRPP	7	/	Excellent
22	Male	23	Falls on ground	LCF	/	Radial	C	Cast	8	Cubitus varus	Excellent
23	Female	22	Falls on ground	Elbow dislocation	/	Ulnar	B	CRPP	96	Cubitus varus	Excellent
24	Male	31	Falls on ground	Elbow dislocation	/	Ulnar	B	CRPP	10	/	Excellent
25	Male	14	Falls on ground	Elbow dislocation	/	Ulnar	A	Cast	5	/	Excellent

SCF = supracondylar fracture of the humerus; LCF = lateral condyle fracture; MCF = medial condyle fracture; CRPP = closed reduction with percutaneous pinning; Combined diagnoses are reported as recorded at first presentation (e.g., MCF + elbow dislocation).

## Data Availability

The data that support the findings of this study are available from the corresponding author upon reasonable request.

## Abbreviations

TFDH	Transphyseal fracture of the distal humerus
CRPP	Closed reduction with percutaneous pinning
ORIF	Open reduction with internal fixation
MEPI	Mayo Elbow Performance Index
SCF	Supracondylar fracture
LCF	Lateral condyle fracture
MCF	Medial condyle fracture
CR	Closed reduction
NBPP	Neonatal brachial plexus palsy
NAI	Non-accidental injury
